# Comparing the Quality of Crowdsourced Data Contributed by Expert and Non-Experts

**DOI:** 10.1371/journal.pone.0069958

**Published:** 2013-07-31

**Authors:** Linda See, Alexis Comber, Carl Salk, Steffen Fritz, Marijn van der Velde, Christoph Perger, Christian Schill, Ian McCallum, Florian Kraxner, Michael Obersteiner

**Affiliations:** 1 International Institute for Applied Systems Analysis, Ecosystem Services and Management Program, Laxenburg, Austria; 2 Department of Geography, University of Leicester, Leicester, United Kingdom; 3 Institute of Behavioral Science, University of Colorado, Boulder, Colorado, United States of America; 4 University of Applied Sciences, Wiener Neustadt, Austria; 5 Remote Sensing and Land Information Systems, Albert-Ludwig University, Freiburg, Germany; University of Warwick, United Kingdom

## Abstract

There is currently a lack of in-situ environmental data for the calibration and validation of remotely sensed products and for the development and verification of models. Crowdsourcing is increasingly being seen as one potentially powerful way of increasing the supply of in-situ data but there are a number of concerns over the subsequent use of the data, in particular over data quality. This paper examined crowdsourced data from the Geo-Wiki crowdsourcing tool for land cover validation to determine whether there were significant differences in quality between the answers provided by experts and non-experts in the domain of remote sensing and therefore the extent to which crowdsourced data describing human impact and land cover can be used in further scientific research. The results showed that there was little difference between experts and non-experts in identifying human impact although results varied by land cover while experts were better than non-experts in identifying the land cover type. This suggests the need to create training materials with more examples in those areas where difficulties in identification were encountered, and to offer some method for contributors to reflect on the information they contribute, perhaps by feeding back the evaluations of their contributed data or by making additional training materials available. Accuracies were also found to be higher when the volunteers were more consistent in their responses at a given location and when they indicated higher confidence, which suggests that these additional pieces of information could be used in the development of robust measures of quality in the future.

## Introduction

The proliferation of Web2.0 technology over the last decade has resulted in changes in the way that data are created. Individual citizens now provide vast amounts of information to websites and online databases, much of which is spatially referenced. The analysis and exploitation of this georeferenced subset of crowdsourced data, or what is more commonly referred to as volunteered geographic information (VGI) [1], [2], has the potential to fundamentally change the nature of scientific investigation. Citizens have a long history of being involved in scientific research or the more recently coined ‘citizen science’ [3]. There are many successful examples of citizen science that have led to new scientific discoveries, including unravelling protein structures [4] and discovering new galaxies [5], as well as websites for public reporting of illegal logging/deforestation [6] and waste dumping [7], which have demonstrated how citizens can have a visible impact upon the environment and local governance. Analysis of more passive sources of geo-tagged data from the crowd from search engines such as Google has also revealed interesting scientific trends, e.g. the relationship between GDP and searches about the future [8], trends in influenza [9] and the ability to characterize crop planting dates [10]. One of the critical advantages of VGI is the potential increase in the volumes of data about all kinds of spatially referenced phenomena. Such data can be collated and used for many different scientific activities: from the calibration of scientific models (e.g. economic prediction models that require information about land use) to the validation of existent data (e.g. maps derived through Earth Observation).

With improved connectivity via mobile phones and the use of low cost, ubiquitous sensors (e.g. those which directly and instantaneously capture data about their immediate environment), the opportunities to exploit such rich veins of VGI are many and varied. However, whilst one of the pressing challenges concerns how to manage large data volumes in terms of processing and storage, a number of yet unaddressed issues persist. These include how to handle data privacy, how to ensure adequate security, and critically, how to assess VGI data quality. Data quality is an area that has attracted increasing attention in the literature [1], [11]–[13]: quantifying VGI data quality underpins its usefulness (that is, its reliability and credibility) and potential for incorporation into scientific analyses. The critical issue is whether ordinary citizens can provide information that is of high enough quality to be used in formal scientific investigations.

With open access to high resolution satellite imagery through providers such as Google Earth and Bing Maps, it is possible to collect vast amounts of volunteered information about the Earth’s surface such as land cover and land use. The collection of crowdsourced land cover data is the main aim of the Geo-Wiki project [14], [15] in what is currently a contributory approach to citizen science [16]. Geo-Wiki is a web-based geospatial portal (http://www.geo-wiki.org) with an interface linked to Google Earth. It can be used to visualize and validate global land cover datasets such as GLC-2000, MODIS and GlobCover [12] which frequently disagree over the land cover they record at any given location [17]–[19]. Since its inception, a number of Geo-Wiki branches have been initiated, each one specifically devoted to gathering different types of information such as agriculture (agriculture.geo-wiki.org), urban areas (cities.geo-wiki.org), biomass (biomass.geo-wiki.org) and more recently human impact (humanimpact.geo-wiki.org).

The general aim of this paper is to determine whether there are significant differences in quality in the information contributed by experts and non-experts. This is explored through a land cover case study with obvious implications for the domains of remote sensing and landscape analyses and investigation of the extent to which VGI can be trusted as a source of training and validation data in remote sensing. However, by investigating generic research questions related to the quality and reliability of information contributed by citizens with different levels of domain expertise, this research should also be of interest to the broader field of citizen science. The next section describes data collection via the human impact Geo-Wiki campaign and the analysis of volunteer and volunteered data quality. Following the results, some discussion is provided regarding the implications of incorporating VGI in scientific research including recommendations for further research before conclusions are drawn in the final section.

## Materials and Methods

### Data from the Human Impact Competition

Crowdsourced data on land cover were collected using a branch of Geo-Wiki called Human Impact (http://humanimpact.geo-wiki.org) and the data were subsequently used to validate a map of land availability for biofuel production [20]. The volunteers were presented with pixel outlines of 1 km resolution (at the equator) projected onto Google Earth (where pixels in this context refer to the smallest area for which information is collected) and were then asked to determine the percentage of human impact and the land cover type at each location from the following list: (1) Tree cover, (2) Shrub cover, (3) Herbaceous vegetation/Grassland, (4) Cultivated and managed, (5) Mosaic of cultivated and managed/natural vegetation, (6) Flooded/wetland, (7) Urban, (8) Snow and ice, (9) Barren and (10) Open Water. The concept of ‘human impact’ was defined as the amount of evidence of human activity visible in the Google Earth images. A spectrum of these intensities is shown in Table 1, which is loosely based on the ideas of Theobald [21]. Volunteers were also asked to indicate their confidence in the class type and the impact score, whether they had used high resolution imagery and the date of the image.

**Table 1:** The spectrum of human impact.

Human Impact	Description
0%	No evidence of any human activity visible
1 to 50%	Some visible evidence of human activities such as tracks/roads; evidence of managed forests; some evidence of deforestation; some scattered human dwellings, some scattered agricultural fields; some evidence of grazing
51% to 80%	Increasing density of agriculture from subsistence on the lower end to intensive, commercial agriculture with large field sizes on the upper end
81% to 99%	Urban areas with decreasing amounts of green space and increasing density of housing
100%	A built up urban area with no green space, typically the business district of a city

Volunteers were recruited by emails sent to registered Geo-Wiki volunteers, relevant mailing lists and contacts, in particular those with students, and through social media. Background information on the competitors was collected through the registration procedure. The competition ran for just under 2 months in the autumn of 2011 [22]. The top ten volunteers were offered co-authorship on a paper resulting from the competition [20] as well as Amazon vouchers as an incentive. Other incentives included inviting friends, which resulted in extra points, a leader board so that competitors could gauge the competition, and appealing to the environmental motivation of individuals through the biofuel theme.

A set of 299 ‘control’ points was used to determine quality where three experts with backgrounds in physical geography, geospatial sciences, remote sensing and image classification agreed upon the land cover at each location. The first 99 control points were provided to the volunteers at the start of the competition, the next 100 were provided three-quarters of the way through and the final 100 were provided at the end, where the latter were drawn from higher resolution imagery. The volunteers were then ranked by an index that combined quality and quantity through equal weighting, and the top ten were declared the winners. Interestingly, there were some minor changes in the top ten once quality was considered.

**Figure 1:**
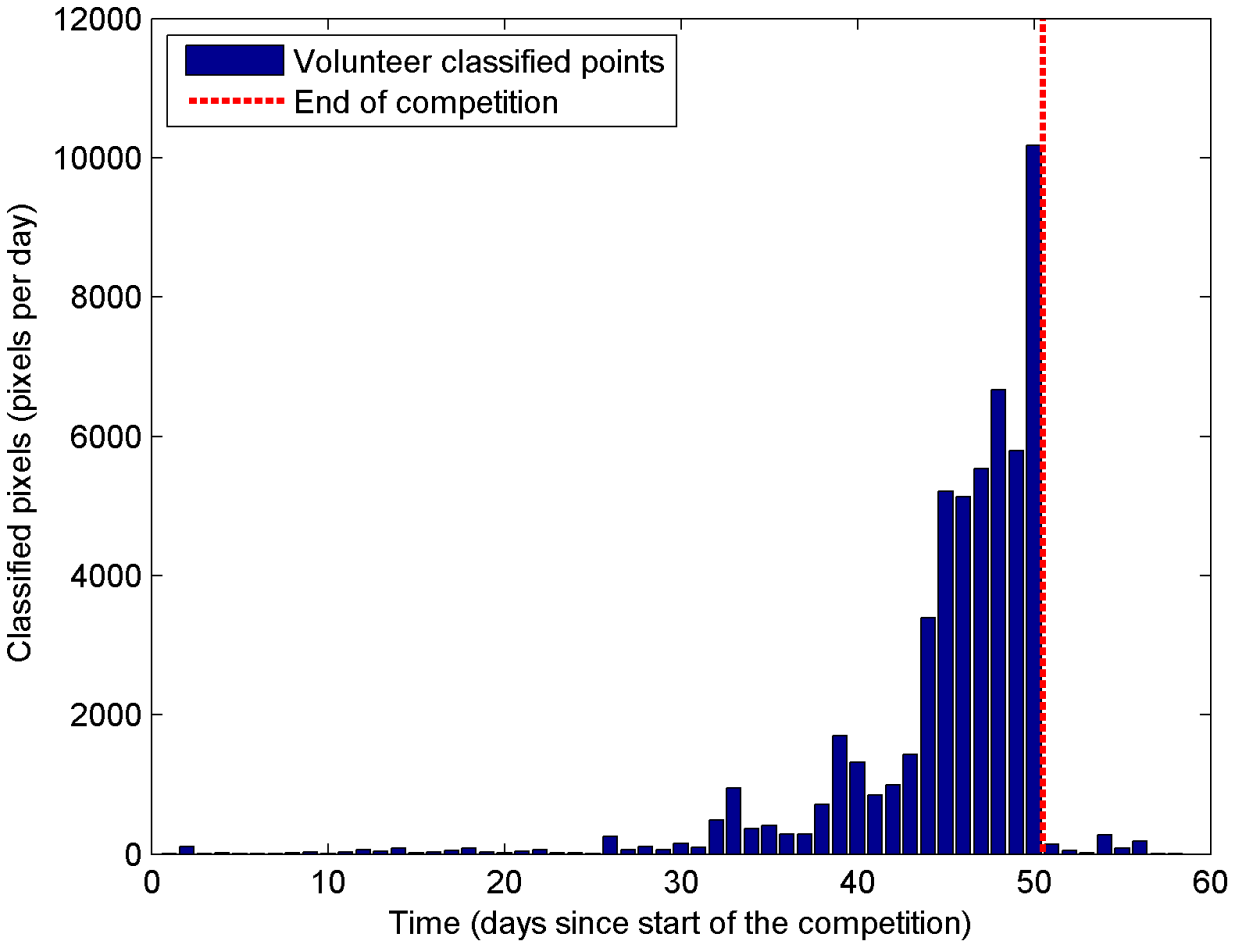
Number of pixels classified per day by the volunteers. These are daily totals from the start of the competition on day 1 to the end at just over 50 days, which shows a clear acceleration as the competition progressed.

**Figure 2:**
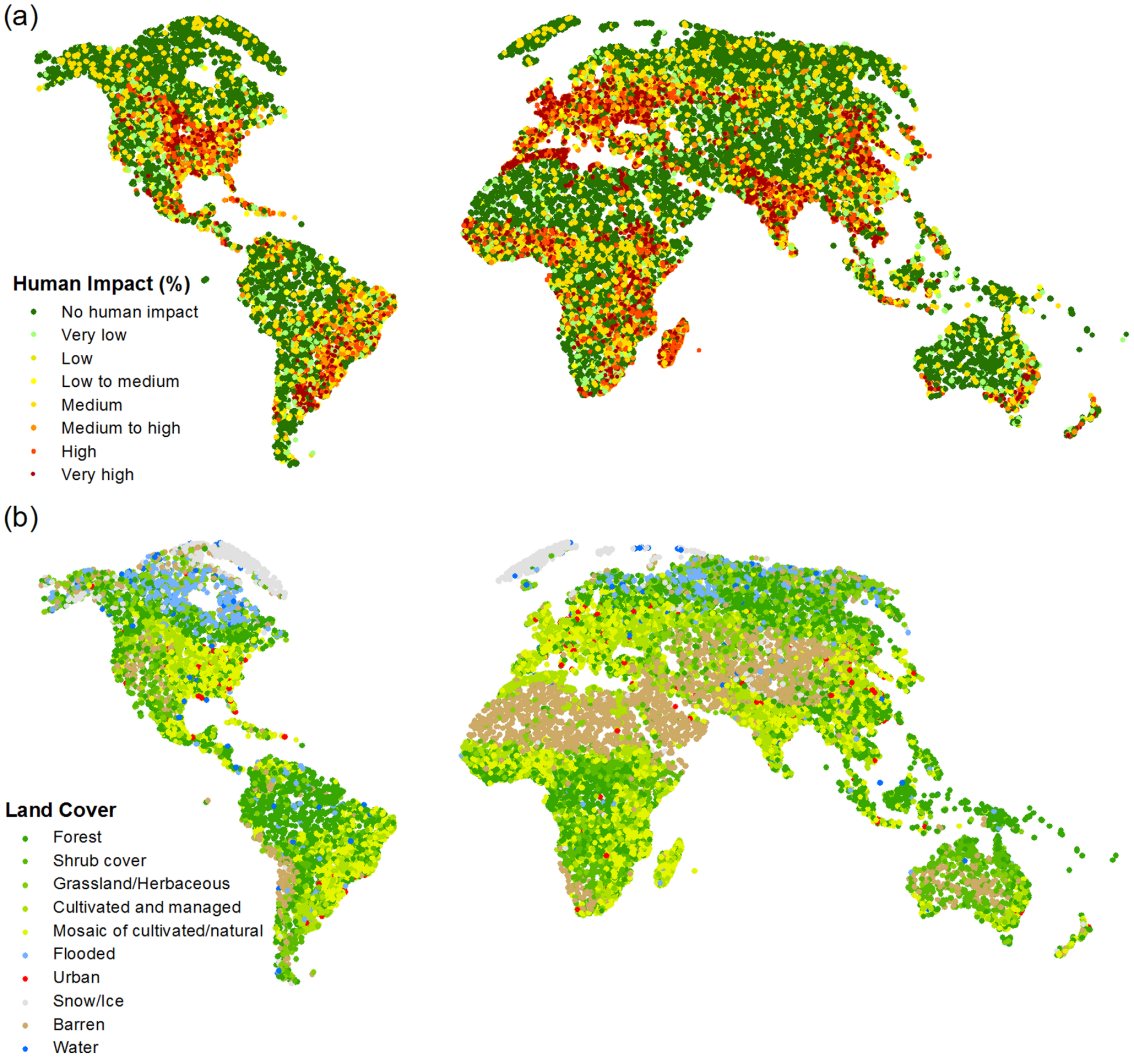
Global distribution of pixels collected by the volunteers. The distribution is shown by (a) human impact and (b) land cover type.

A total of ∼53,000 locations were validated by more than 60 individuals and Figure 1 shows the rapid increase in contributions in the last 20 days of the competition, with a particularly large spike at the end. Figure 2 illustrates the spatial distribution of the ∼53,000 points collected expressed as measures of human impact and land cover. Note that the crowdsourced data can be freely downloaded from http://www.geo-wiki.org.

Of these ∼53,000 validations, 7657 were at the control locations, which were then used to assess quality. The data were then filtered for ‘unknown’ expertise resulting in 4020 control data points scored by 29 Expert volunteers and 3548 control data points scored by 33 Non-expert volunteers. Experts were considered to be individuals with a background in remote sensing/spatial sciences versus non-experts who were new to this discipline or had some self-declared limited background. The control data, whose analysis forms the basis of the paper, have the following characteristics. Experts evaluated an average of 64.8 control data points each (s.d. 108.1) and non-experts 57.2 (s.d. 95.1). Although there is the potential for a few individuals to have a disproportionately large impact on data quality and composition, in this case, of the 29 experts, 18 contributed more than 50 evaluations, and of the 33 non-experts, 19 evaluated more than 50 data points. The volunteers’ demographics (age, gender, socio-economic status etc.) were not captured as part of the contributor registration. This is unfortunate, because although a proxy for previous experience is evaluated in this paper, it is well recognised that such factors can influence contributor responses. Such data will be collected in future campaigns.

### Analysis of Human Impact

To determine how well the answers provided by the volunteers matched the control data in terms of the degree of human impact, a linear regression was fit as follows:
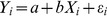
(1)where *Y_i_* is the degree of human impact from the control data, *X_i_* is the degree of human impact from the volunteers, *a* and *b* are coefficients of the linear regression equation and *ε_i_* is a normally distributed random error term for each observation *i*.

Each volunteer provided information on expertise during registration. Equation 1 was extended to include an indicator of respondent expertise in the regression model:
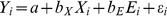
(2)where, in addition to the previously defined variables, *b_X_* is the regression coefficient for volunteer human impact, *E_i_* is the expertise indicator variable for observation *i* (0 for Non-Expert, 1 for Expert), and *b_E_* is the regression coefficient for this variable. Thus, this coefficient is a measure of the difference in human impact (on aggregate) between the Non-Expert and Expert contributions. This model implicitly assumes human impact is equally predicted by experts and non-experts (i.e. is uniform), and assumes a uniformity of the intercept term within each expert group, if the intercept is considered to be *a* for the non-expert group, and *a*+*b_E_* for the expert one.

The data provided by the volunteers were then analysed for consistency, which is a known issue in ground truthing [23]. After every 50 points, the volunteers were provided with a point they had previously validated. The average, median and standard deviation of the maximum difference between the volunteers and the controls were calculated for all control points, by expertise, by volunteer consistency in the land cover they recorded, and by confidence.

**Figure 3:**
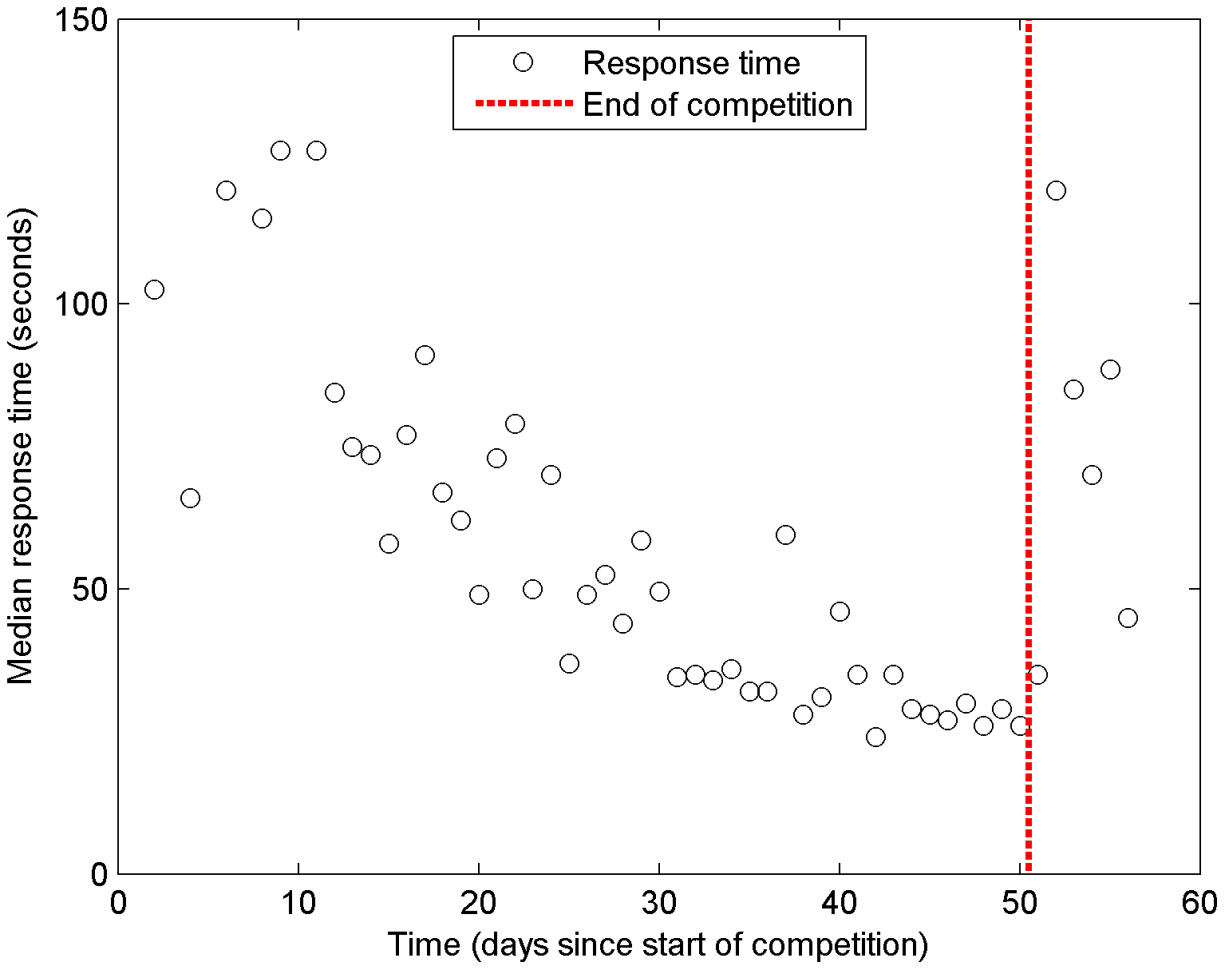
Median response time of the volunteers. The response time is in seconds measured from the start of the competition until the end at just over 50 days.

Finally, the response times of the volunteers were calculated between each successive data point they scored. The median response time was 55 secs with a first and third quartile of 32 and 100 secs respectively. The average response time was 5,226 secs, indicating a highly skewed distribution, which reflects large pauses in contributions, e.g. at the end of a validation session. Figure 3 shows the median response time per day over the course of the competition. There is a general trend towards shorter response times as the competition unfolded with the shortest response times between successive validations occurring at the end of the competition. Thus, we were interested in understanding the relationship between response time and quality of the human impact responses overall and whether there was any difference in quality towards the end of the competition.

The response time data were first pre-processed in two ways. First, all response times greater than 5 minutes were removed as these were deemed unrepresentative of typical behavior. This was based on visual inspection of the distribution. However, 5 minutes also represents the 92.5^th^ percentile and therefore includes the majority of the data. Second, response times were log transformed due to the skewness of the distribution. A linear regression equation of the form given in (1) was fit to the entire dataset where the dependent variable, *Y_i_*, was the absolute difference in the answers for human impact between the control data and the volunteers’ scores, and the independent variable, *X_i_,* was the log of the response times, with *a* and *b* representing coefficients of the linear regression, and *ε_i_* the error term for each observation *i*.

The last 100 control points provided to the volunteers at the end of the competition were locations of cropland or agricultural land covers (the classes of *Cultivated and managed* and *Mosaic of cultivated and managed/natural vegetation*) and where high resolution images existed. In order to evaluate how volunteer performance changed with experience, only control points with agricultural land cover and where high resolution images were available were selected from the first 199 control points. The average accuracy in human impact across the first two control sets was then compared to the average accuracy of the third set using a t-test to determine whether there were any significant differences.

### Analysis of Land Cover

As in the analysis of human impact scores above, control points were used to evaluate volunteer accuracy in terms of the land cover they indicated. An error or confusion matrix was populated for all contributors (Table 2) and the overall accuracy was calculated as follows:
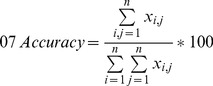
(3)where *i* is the volunteer class, *j* is the control class and *n* is the total number of classes.

**Table 2:** A confusion matrix for the comparison of controls with responses from the crowd.

	Class 1 (control *j*)	Class 2 (control *j*)	…	Class *n* (control *j*)
Class 1 (volunteer *i*)	*x_1,1_*	*x_1,2_*	*…*	*x_n,1_*
Class 2 (volunteer *i*)	*x_2,1_*	*x_2,2_*	*…*	*x_n,2_*
…	*…*	*…*	*…*	*…*
Class n (volunteer *i*)	*x_n,1_*	*x_n,2_*	*…*	

In addition, two other measures of accuracy were calculated, specific to each land cover class: user’s and producer’s accuracies. User’s accuracy describes errors of commission or Type I errors. For example, the user’s accuracy for the forest class indicates the likelihood that what was labeled as forest by the volunteers really is forest. Producer’s accuracy reflects errors of omission or Type II errors. Using the forest example again, this measure reflects how well the forest cover control pixels were classified by the volunteers. These two measures are calculated as follows:
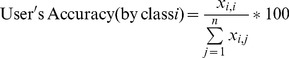
(4)

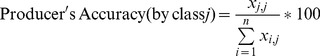
(5)where *i* is the volunteer class, *j* is the control class and *n* is the total number of classes.

Separate accuracy measures were calculated for the three sets of control pixels (to determine whether accuracies change over time) for locations where the volunteers were the most confident and to compare experts and non-experts.

Contributor consistency in land cover labeling was then analysed by determining the proportion of times when the same land cover type was chosen when presented with the same data point. This was calculated for all points, by expertise, and by various degrees of confidence.

Finally, the impact of response time on the quality of land cover validations was analysed using logistic regression of the following form:
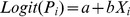
(6)where the probability (*P_i_*) that the land cover is correctly identified is expressed as a function of response time, *X_i_*.

The effect of response time on accuracy in the final set of controls was compared with the first and second set to determine whether contributors were more interested in scoring a greater number of points and spent less time on each data point towards the end of the competition. A two-tailed binomial test was used to test whether the number of correct classifications at the end of the competition was greater than expected based on the total number of classifications performed and the probability of correct classification in the earlier part of the competition.

## Results and Discussion

### Human Impact

The result of the regression described in Equation 1 to determine how well the degree of human impact can be predicted by the contributors based on the control points is provided in Table 3. This shows that *b* differs significantly from zero and is positive but less than 1 suggesting that there is evidence that the users underestimated the degree of human impact by roughly 30 percent.

**Table 3:** Regression analysis for the model *Y_i_ = a+bX_i_+ε_i_*, where *Y_i_* is the degree of human impact from the control data, *X_i_* is the degree of human impact from the participants.

	Estimate	Std. Error	t value	Pr(>|t|)
*a*	11.300	0.363	31.16	0.000
*b*	0.699	0.006	122.43	0.000

The results of including an indicator variable describing respondent expertise (Equation 2) are shown in Table 4. The slopes are still positive and suggest that allowing for expertise even in a simple way changes the results of relating to the slope term. To investigate this further, Equation 1 was extended to include variables describing expertise. Although computed together, this effectively splits the regression into two models - one for each of the expert groups - and the results are shown in Table 5. These results indicate that there is little variation in the degree to which the expert and non-expert group underestimated the degree of human impact.

**Table 4:** Extending the regression to include an indicator of expertise, where *b_E_* is the regression coefficient for this indicator and *b_X_* is the regression coefficient for participant human impact scores.

	Estimate	Std. Error	t value	Pr(>|t|)
*a*	9.009	0.432	20.85	0.000
*b_X_*	0.705	0.006	123.49	0.000
*b_E_*	4.251	0.442	9.62	0.000

**Table 5:** The regression analysis of predicting the degree of human impact by expert and non-expert groups, when the regression is split into 2 simultaneous models.

	Estimate	Std. Error	t value	Pr(>|t|)
*a (Expert)*	7.960	0.527	15.12	0.000
*a (Non-expert)*	14.200	0.494	28.74	0.000
*b (Expert)*	0.725	0.008	91.06	0.000
*b (Non-expert)*	0.685	0.008	83.61	0.000

**Figure 4:**
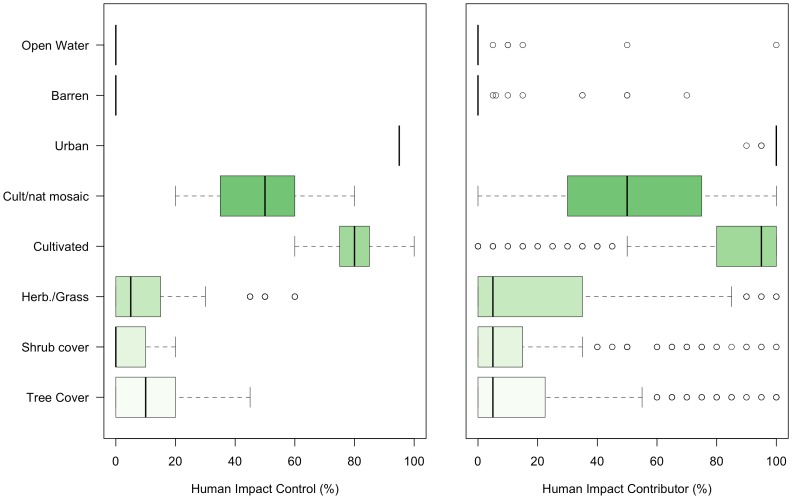
The distribution of human impact by land cover class. The distribution is shown for (a) the control pixels and (b) the volunteers, where the latter show a much wider range of answers.

Figure 4 shows the distribution of human impact scores for the control pixels and the contributor data by land cover class. It shows a general trend for contributors to underestimate the degree of human impact across the different land cover types with the exception of *(5) Mosaic of cultivated and managed/natural vegetation*.

**Figure 5:**
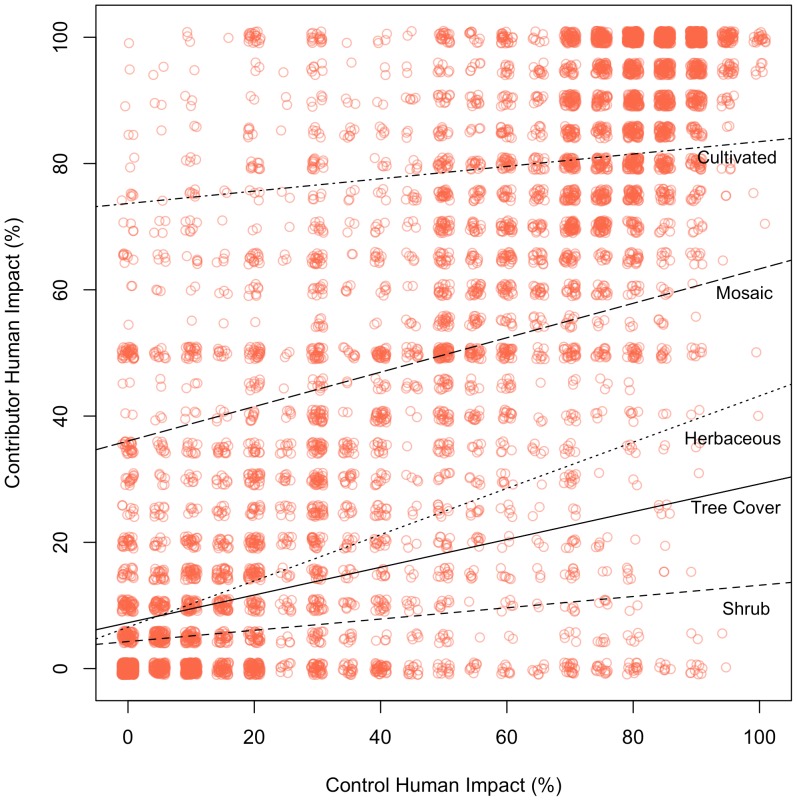
The relationship between the volunteer responses and the controls for human impact by land cover type. The lines show the coefficient slopes when each control land cover class is evaluated in turn. Note that the data points have had a small random noise component added to allow their density to be visualised.

A further analysis explored how human impact scores varied with land cover class. The standard regression described in Equation 1 was extended to include indicators for the land cover classes. Since there was only a small number of data points classified as *Open water*, *Barren* or *Urban*, these classes were excluded from the regression analysis. The results for the remaining five land cover types are shown in Table 6 and Figure 5 plots the contributed against the control human impact scores with the regression coefficients for different land cover classes.

**Table 6:** Regression analysis for the degree of human impact.

	Estimate	Std. Error	t value	Pr(>|t|)
*a (Tree cover)*	7.264	0.343	21.16	0.000
*a (Shrub cover)*	4.284	0.520	8.24	0.000
*a (Herb./Grass)*	6.567	0.504	13.03	0.000
*a (Cultivated)*	73.669	0.857	86.01	0.000
*a (Cult./nat mosaic)*	36.046	0.485	74.32	0.000
*b (Tree cover)*	0.220	0.012	18.52	0.000
*b (Shrub cover)*	0.089	0.021	4.34	0.000
*b (Herb./Grass)*	0.366	0.015	24.62	0.000
*b (Cultivated)*	0.098	0.010	10.06	0.000
*b (Cult./nat mosaic)*	0.273	0.008	33.58	0.000

The results show that the prediction of the degree of human impact varies with land cover classes. The coefficients for the *Herbaceous vegetation/Grassland* class most strongly predict human impact, the coefficients for the *Shrub cover* class are the weakest predictors and all classes underestimate human impact. This indicates that the conceptualizations of these classes may need to be more clearly defined and perhaps more training examples used to illustrate the different degrees of human impact by land cover type.

Table 7 shows the results of the consistency analysis. Overall the contributors were consistent in their answers regarding the degree of human impact, with an average deviation of less than 10% (i.e. 9.6%) although the spread of answers was higher at 17.4%. When expertise was considered, non-experts had a lower average deviation than the experts by just under 3%. When the consistency was extended to land cover, those pixels which showed consistent choices in land cover had a lower average deviation in human impact by 8.3% compared to those which showed inconsistency in land cover choice. This reflects pixels that were clearly more difficult to identify. Finally, when contributors were the most confident in their choice of human impact, they were also more consistent (average deviation of 7.9%), with consistency decreasing as confidence decreased resulting in an average deviation of as much as 25.9% for the least confident category. This analysis of consistency serves to highlight the need to examine those pixels which were not consistently labeled and which are probably more difficult to judge in terms of both human impact and land cover, which can then be used to help train the volunteers.

**Table 7:** Consistency of response to degree of human impact.

Disaggregation	Category	Average HI (%)	Median HI (%)	Std Dev (%)
All	All points	9.60	0.00	17.43
Expertise	Experts	10.90	5.00	18.50
	Non-experts	7.95	0.00	15.82
Land cover consistency	Agree on land cover between points	7.20	0.00	14.55
	Disagree on land cover between points	17.25	10.00	22.80
Confidence	Sure	7.92	0.00	15.68
	Sure+Quite sure	9.13	0.00	16.93
	Quite sure+Less sure+Unsure	22.08	15.00	23.65
	Less sure+Unsure	25.92	15.00	25.16

The results of the regression analyzing the effect of response times are shown in Table 8 and indicate that the agreement between the volunteers and the control pixels increased significantly with a faster response time for human impact, although the effects were small. For each increase in magnitude in response time, the agreement between the crowd and the control pixels increased in accuracy by 1.4%. The average deviation in human impact for pixels of *(4) Cultivated and managed* and *(5) Mosaic of cultivated and managed/natural vegetation* and high resolution imagery from the first two control sets was 17.1%. This was compared to the third set of control data points (consisting of only these pixel types) and the average deviation in human impact was lower, decreasing to 14.7%. A t-test confirmed that the means are significantly different from one another (p<0.0001; t = −4.8533; degrees of freedom  = 3326.222) and showed that accuracy in human impact actually increased at the end of the competition. Thus, these analyses indicate that there are no particular concerns over quality in relation to response time.

**Table 8:** Regression analysis for the model *Y_i_ = a+bX_i_+ε_i_* where *X_i_* is response time and *Y_i_* is human impact.

	Estimate	Std. Error	t value	Pr(>|t|)
*a*	12.9915	1.0706	12.135	0.000
*b*	1.4110	0.6157	2.291	0.022

### Land Cover

The overall accuracies for the three sets of control points labeled C1, C2 and C3 are presented in Table 9 for the full dataset, considering only those contributions where confidence was high (i.e. ‘sure’ on the slider bar) and then disaggregated by expertise (i.e. experts or non-experts).

**Table 9:** Accuracy of land cover (in %) based on comparison of volunteer response with three sets of controls.

Dataset used	No allowance for confusion between classes		
	C1	C2	C3
Full dataset	66.4	66.5	76.2
Confidence rating of sure	69.4	69.3	78.9
Experts	69.2	72.3	84.6
Non-experts	62.4	61.9	65.9

Considering all three sets of control data, accuracy varies between 66 and 76%. There is little difference between the first and second set of controls but there is a marked increase in accuracy for the final set (C3) with 76%. This is unsurprising since the final control sample was drawn from high resolution imagery. When taking only those answers where the volunteers indicated high confidence (or ‘sure’ on the slider bar), there was around a 3% increase in the accuracy to 69%. Unlike with human impact, experts were more accurate than non-experts, e.g. 62% for non-experts and 69% for experts for C1 with even larger differences observed for C2 and C3. This suggests that extra training should be provided to those individuals with a non-expert background. As training manuals are often unread or rarely consulted, a more interactive approach could be introduced such that the volunteers are made aware of their errors as they progress through a competition. In addition, a forum could be set up to discuss pixels that present difficulties in identification, particularly for non-experts.

Table 10 shows the user’s and producer’s accuracies for the five most common land cover types in the dataset. Overall the results show that there is generally an increase in the accuracy across control sets although C3 should only really be considered for cropland and mosaic classes. The lowest accuracies are in shrub cover, grassland/herbaceous and the mosaic cropland class, which indicates the need to provide more examples of how these classes appear on Google Earth within the training materials as the volunteers are confusing these classes more often than others. When considering points where the volunteer had a high confidence, the patterns are similar and there is generally an increase in accuracy although the mosaic cropland class continues to be more problematic, with a decrease in the user’s accuracy across control sets. Finally, the effect of expertise on land cover classification accuracy produced variable results depending upon the land cover type and the control set considered. For the forest class, the non-experts improved in their ability to correctly identify forest by the second set of controls, while the non-experts actually showed a decrease in the producer’s accuracy. Similarly, for the shrub class, the non-expert showed a greater level of improvement in the second set of controls compared to the expert and outperformed them in terms of both user’s and producer’s accuracy in C2. The experts were better than non-experts at identifying herbaceous, cropland and mosaic but once again there were differences in the user’s and producer’s accuracies. By building up a picture of where experts and non-experts have differing performance by land cover class, we can tailor the kinds of training materials provided to the volunteers, focusing on areas where greater problems in identification lie.

**Table 10:** User’s and producer’s accuracies for the five main land cover types and for different subsets of the data including confidence and expertise.

Data set	Land cover type	No confusion					
		User’s accuracy			Producer’s accuracy		
		C1	C2	C3	C1	C2	C3
Full	1	75.9	77.4	43.6	67.1	69.6	100.0
	2	52.1	46.5	0.0	61.7	67.2	N/A
	3	45.1	56.3	6.0	51.3	56.3	30.0
	4	78.9	88.8	95.2	74.2	72.8	76.0
	5	71.5	68.8	64.6	62.2	60.7	76.4
Sure	1	78.7	82.4	53.1	68.0	70.2	100.0
	2	50.8	48.6	0.0	64.4	71.2	N/A
	3	43.9	52.4	10.7	47.7	53.7	50.0
	4	81.0	89.6	95.2	76.5	75.0	78.7
	5	72.4	68.2	63.7	66.8	65.8	78.8
Expert	1	78.4	83.5	52.6	73.0	68.8	100.0
	2	54.8	45.7	0.0	63.8	65.1	N/A
	3	50.9	65.6	7.1	52.4	65.2	33.3
	4	77.1	90.5	95.5	82.6	80.5	86.5
	5	76.5	75.7	78.1	59.3	71.8	80.2
Non-expert	1	71.9	73.6	35.0	58.6	70.2	100.0
	2	48.5	47.2	0.0	58.9	68.8	N/A
	3	38.0	48.7	5.6	49.5	48.9	28.6
	4	82.8	87.0	94.6	61.2	66.3	63.0
	5	66.1	62.4	52.5	66.3	51.6	71.8

1 =  Tree cover; 2 =  Shrub cover; 3 =  Herbaceous vegetation/Grassland; 4 =  Cultivated and managed; 5 =  Mosaic of cultivated and managed/natural vegetation.

Similar to human impact, a further analysis was then undertaken on a subset of the data where the volunteers were provided with the same pixels at different times in the competition (Table 11). The results show that the volunteers were consistent in their response just over 76.1% of the time where this was slightly lower for experts (75.7%) and slightly higher for non-experts (76.7%). A very minor increase to 77.6% was observed when considering only those pixels where the volunteer was sure but when the volunteers were less sure or unsure about their responses, their consistency in response decreased to 66.7%.

**Table 11:** Consistency of response in choosing the land cover type.

Disaggregation	Category	Consistent	Percentage
None	Full dataset	Y	76.1
		N	23.9
Expertise	Expert	Y	75.7
		N	24.3
	Non-Expert	Y	76.7
		N	23.3
Confidence	Sure	Y	77.6
		N	22.4
	Quite sure+Lesssure+Unsure	Y	76.4
		N	23.6
	Less sure+Unsure	Y	66.7
		N	33.3

The final analysis concerned the relationship between quality in land cover classification and response time. The results showed that the crowd was 40% more likely to disagree with the control for each order of magnitude increase in response time (p<.0001) as shown in Table 12 and indicated by the value of *b*.

**Table 12:** Logistic regression analysis for the model *Logit* (*P_i_*)* = a+bX_i_* where *X_i_* is the log of the response time and *P_i_* is the probability that the land cover is correctly identified.

	Estimate	Std. Error	t value	Pr(>|t|)
*a*	1.46573	0.13955	10.504	0.000
*b*	−0.40005	0.07957	−5.027	0.000

Considering the issue of whether quality in land cover validation (and therefore accuracy) decreased near the end of the competition, we compared the probability that the volunteers agreed with the control pixels for land cover types (4) *Cultivated and managed* and (5) *Mosaic of cultivated and managed/natural vegetation* at the end of the competition (75.9%) with that from the early to middle part of the competition (70.6%). This difference was determined to be highly significant (p<.0001; number of trials  = 1500; number of successes  = 1139) using a binomial test and therefore the accuracy in estimating land cover actually increased in the final stages of the competition. Thus for both human impact and land cover, there are no concerns about the quality decreasing near the end of the competition with a faster response time.

### Conclusions

This paper assessed the quality of crowdsourced data collected through a Geo-Wiki competition. Volunteers identified the degree of human impact and classified land cover at random locations using Google Earth images. Quality was assessed by comparing volunteer results with results agreed by experts at a number of control points. Control points were provided to volunteers at the beginning, middle and end of the competition. The results showed that there is little difference between experts and non-experts in identifying human impact while experts were better than non-experts in identifying land cover. However, the results for both varied by land cover type and through the competition. For example, experts were better than non-experts at identifying shrub land cover at the start of the competition but non-experts improved more than experts and then outperformed them in shrub cover identification by the middle of the competition, indicating that volunteers were learning over time. The volunteers were shown to be reasonably consistent in their characterizations of human impact and land cover with non-experts outperforming the experts in terms of human impact and vice versa for land cover. Moreover, when contributors were confident in their choice of human impact, they were also more consistent, and unsurprisingly, consistency decreased as confidence decreased. Finally, increased response times (as observed towards the end of the competition) did not have a negative impact on quality, and volunteers were therefore not sacrificing quality for the desire to complete more locations and thereby win the Geo-Wiki competition. Thus overall, the non-experts were as reliable in what they identified as the experts were for certain, identifiable situations, and the reliability of the information provided by non-experts improved faster and to a greater degree than experts. Thus, better, targeted training materials and a continual learning process built into the competition might help address these issues. Also, allowing volunteers to reflect on the information they contribute, for example by regularly feeding back evaluations of their data through the use of control points or by making additional material available to them, would also potentially decrease differences between experts and non-experts, particularly in the classification of land cover. The findings of this research relating to the differences between expert and non-expert citizens are also relevant to other areas of research that seek to benefit from the advantages of citizen science. For example, recent activity such as the umbrella Zooniverse project (http://www.zooniverse.org) promotes collaborative projects in many areas of social and physical science research. Currently, registration to its projects captures no information about the contributor, their training or their socio-economic context. Approaches that include information about participant background, control points, reflection, repetition, etc. have broad potential for other citizen science projects that involve classification or identification, e.g. [24], [5] where experts can be used to build a database of controls for monitoring and learning purposes.

The next step in this research is to develop robust measures of quality for each location in the crowdsourced database based on rules that take into account the number of times that contributors have provided information at a given location along with the consensus in the answers, their expertise and the confidence in the answers provided. However, the results from this study suggest the need for more nuanced approaches than a simple Linus Law or mass of evidence approach (which have been previously suggested in this domain) for determining when to believe the crowd and therefore when the information they provide can be used with confidence. Formal methods for combining evidence such as Bayesian probability, Dempster-Shafer theory of evidence, Possibility Theory and Endorsement theory provide different ways for combining or partitioning evidence. They allow measures of certainty and uncertainty to be generated and provide different measures of confidence in aggregated information and for determining when the weight of evidence indicates that crowdsourced data or VGI are ‘believable’. Since the relationship between reliability and confidence was found to be strong in this research, this also suggests that future activities seeking to incorporate crowdsourced data should capture measures of contributor confidence in the information they provide. Ongoing research by the authors will investigate these areas in more detail.
